# Targeting male mosquito mating behaviour for malaria control

**DOI:** 10.1186/s13071-015-0961-8

**Published:** 2015-06-26

**Authors:** Abdoulaye Diabate, Frédéric Tripet

**Affiliations:** Institut de Recherche en Sciences de la Santé/Centre Muraz, Bobo-Dioulasso, Burkina Faso; Centre for Applied Entomology and Parasitology, School of Life Sciences, Keele University, Newcastle-under-Lyme, UK

**Keywords:** *Anopheles gambiae*, Swarm ecology, Mating behaviour, Vector control, Sterile male release, Transgenic mosquito releases, Swarm killing, Lure-and-kill

## Abstract

Malaria vector control relies heavily on the use of Long-Lasting Insecticidal Nets (LLINs) and Indoor Residual Spraying (IRS). These, together with the combined drug administration efforts to control malaria, have reduced the death toll to less than 700,000 deaths/year. This progress has engendered real excitement but the emergence and spread of insecticide resistance is challenging our ability to sustain and consolidate the substantial gains that have been made. Research is required to discover novel vector control tools that can supplement and improve the effectiveness of those currently available. Here, we argue that recent and continuing progress in our understanding of male mating biology is instrumental in the implementation of new approaches based on the release of either conventional sterile or genetically engineered males. Importantly, further knowledge of male biology could also lead to the development of new interventions, such as sound traps and male mass killing in swarms, and contribute to new population sampling tools. We review and discuss recent advances in the behavioural ecology of male mating with an emphasis on the potential applications that can be derived from such knowledge. We also highlight those aspects of male mating ecology that urgently require additional study in the future.

## Review

### Introduction

The landscape of malaria control has dramatically changed over the last few years [1]. In the 2008 report of WHO, the estimated death toll of malaria decreased from about 2 million deaths/year to less than 700,000 [2]. Although several factors might have contributed to this sharp decline, vector control, mainly through Indoor Residual Spraying (IRS) and the mass distribution of Long-Lasting Insecticidal Nets (LLINs), has played a major role [3]. Sadly, the emergence and continuous spread of insecticide resistance is threatening the future of vector control and making it clear that alternative tools will be needed to maintain and consolidate the gain so far accrued. The MalERA Consultative Group in its 2011 report defined the research agenda that would be needed to sustain and improve the effectiveness of currently available control tools. Specifically, emphasis should be placed on developing interventions that affect vector species and populations not effectively targeted by current tools. That entails focusing on other aspects of mosquito biology including outdoor feeding and resting as well as mating behaviour.

For several decades, malaria vector control strategies have primarily focused on female mosquitoes and nobody has ever seriously looked at the possibility of impacting mosquito populations by targeting males (but see [4, 5]). Consequently the agenda of vector research was mostly female-oriented. In 2005 Ferguson *et al.* [4] conducted a web-based literature search and showed that of the 900 papers published on *Anopheles gambiae* from 1980 to 2004, only 19 were relevant to males. The reason for the lack of interest seems obvious. Males do not bite and hence are not disease vectors *per se*, so why then should one bother? In addition, it has not always been easy for those who explored male mating biology to locate swarms in the field. They occur at sunset and only for 20-40 min. Missing this window of time results in an entire day being lost. As of today, many field entomologists have never observed the most deadly malaria vector *An. gambiae* swarming. What does it look like and how can a better understanding of its mating biology contribute to malaria control?

Over the last decade a growing number of studies have focused on the ecology of mating and swarming in *An. gambiae* with three major ultimate objectives in sight: First, to unravel mechanisms of reproductive isolation and conspecific recognition among the sibling species and forms of the *An. gambiae* complex. Secondly, to assess the feasibility of novel vector population control approaches through male swarm killing or trapping that could be integrated into or complement existing vector control programmes. Thirdly, to characterize essential factors that enhance male mating competitiveness as a way of improving mosquito malaria control programmes relying on male mosquito releases. Here, we summarize the advances we feel are most relevant to these endeavours and discuss key practical issues that are essential to translate these findings into improved malaria control tools and strategies.

### Advances in swarm ecology

#### The mating system of *Anopheles gambiae*

**Fig. 1:**
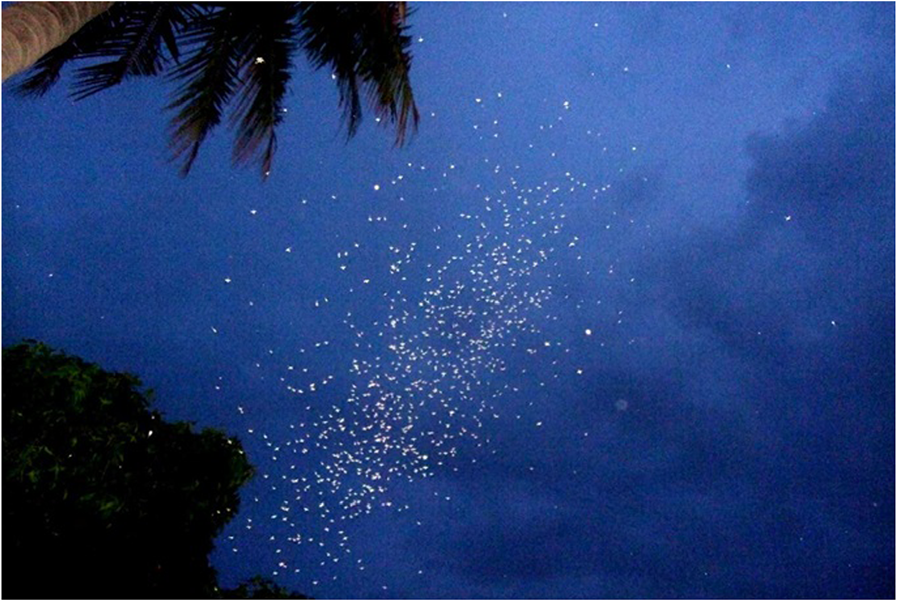
Photograph of *Anopheles gambiae* ss swarm in the VK5 village, Vallée du Kou in Burkina Faso. Little white dots against the blue sky are male mosquitoes swarming and attempting to mate with females that sporadically visit them

*An. gambiae* s.s., like many mosquito species, mates in flight (Fig. 1). Males gather in swarms at specific mating sites over landmarks known as swarm markers ([6] and references therein). It is not known how males are attracted to these landmarks, but visual cues seem to play an important role in selecting the swarming sites. Various markers have been identified in the field including woodpiles, trash piles, wells, intersection of footpath and grasses. While one may wonder how mosquitoes are attracted to so many different objects, all these markers have something in common. They either form a dark-light contrast on the ground or they break down the regularity of a smooth landscape and that discontinuity seems to attract males. *An. coluzzii*, formerly known as the M molecular form of *An. gambiae* [7], exploits most of these various markers while *An. gambiae* s.s., or S molecular form, is mostly found on bare ground. This attraction to conspicuous markers is widespread among mosquitoes [6, 8–10] and across different swarming insect species [11]. The size, shape and height of the swarm above ground are more likely constrained by the immediate landscape of the swarming arena. Mosquito swarms were seen to spread vertically or horizontally given the shape of the swarm markers [12]. In the specific case of the M molecular form, a clear view of the horizon seems to determine the height of the swarms above ground. When the swarm marker was located too close to an object that blocked the view, the swarms would then fly high above the ground to capture the light of the horizon. All of this information suggests that specific stimuli emanating from these markers is captured and processed similarly in mosquito brains and elicits specific behaviour. For example, the same markers are not only used in different species including *An. arabiensis, An. funestus, An. melas* and *An. rufipes*, but also across geographic regions such as Burkina Faso, Mali, Benin, the Gambia, Sudan, Tanzania and Mozambique [6, 8–10, 12].

**Fig. 2:**
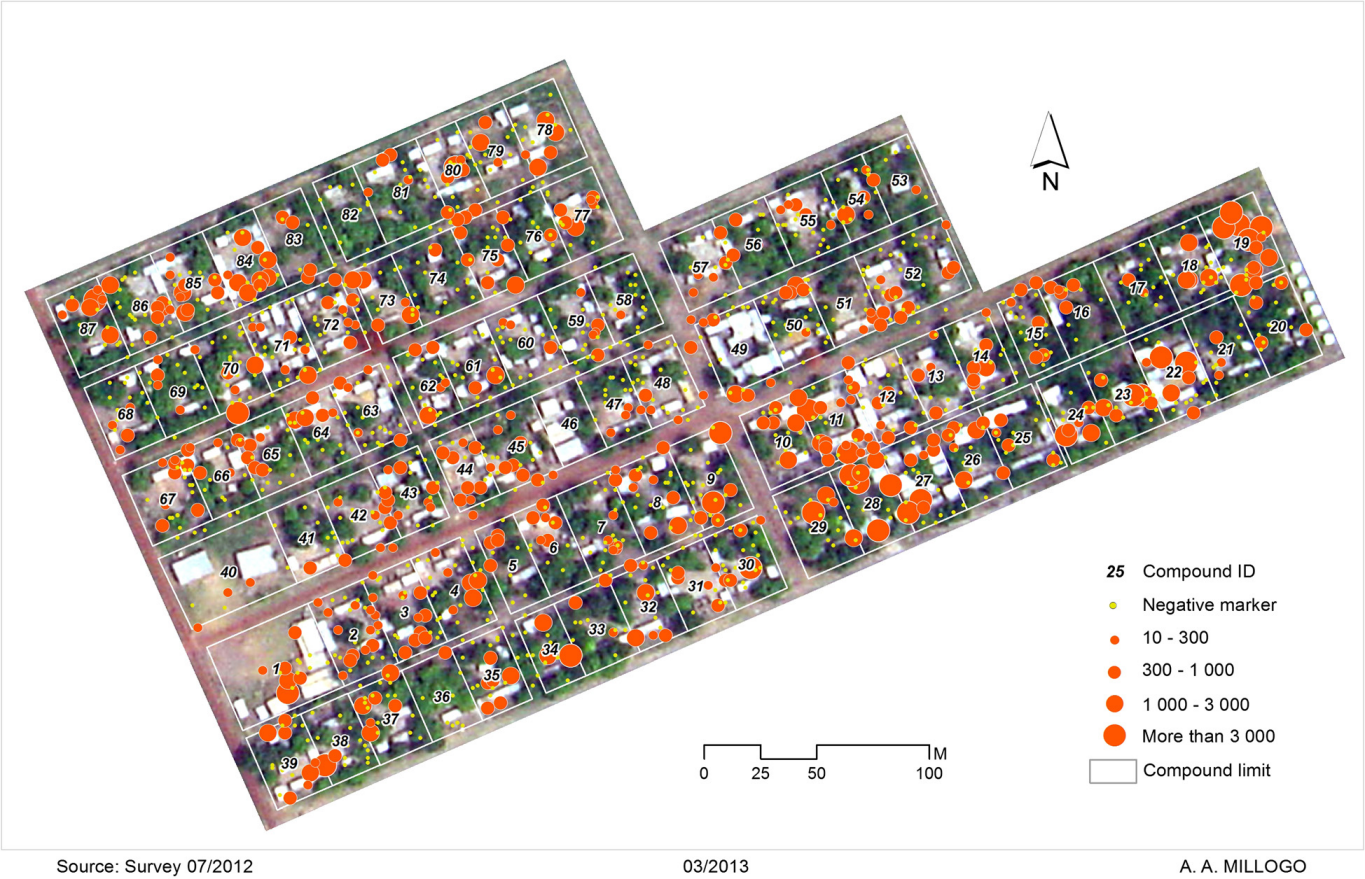
Distribution map of *An. gambiae* swarms in VK5 village, Vallée du Kou. Orange dots represent sites effectively occupied by swarming males and yellow ones represent potential swarming sites that were not occupied. Aggregation of the orange dots indicates that swarms are clustered in space

Swarms are composed of males with females typically entering a swarm and leaving *in copula*. The mating stations contain no resources and females visit these stations solely to copulate. From an evolutionary perspective, the key question is how swarming behaviour has evolved in mosquitoes. In *An. gambiae*, as in many other mosquitoes, females mate only once in their lifetime while males can mate several times. In the long run, the number of mate-seeking males will drastically outnumber that of females. In an environment where female distribution is over-dispersed, it might be beneficial for both sexes, but mostly for males, to show up at specific mating stations and at specific times in order to attract females. This mating system is known as lekking. Among other properties it is characterized by female choice and male display, which in mosquitoes may be constrained by the often large numbers of individuals involved and the limited swarming duration (but see [13] and section below). Hence the mating system of *An. gambiae* has been defined as lek-like but incorporates scramble mating competition characteristics [6]. Three major theoretical models have been put forward to explain it [14]. Of all these models, the hotspot model seems to better reflect the *An. gambiae* swarm. In this model, leks are male-initiated ([14] and references therein). Males set themselves in places where the probability of encountering receptive females is the highest. Males have no direct information about female location, but they use cues from non-defendable resources that females use to set their sites. Consequently, the highest density of males will coincide with the highest density of females. Interestingly, a report on the pattern of swarm spatial distribution indicated that swarms are not randomly distributed across space [6]. A recent investigation over three years in the Valley de Kou, where the highest swarm densities have been observed, indicated a strong clustering pattern of swarms around human habitations (Fig. 2). However, the environmental cues and behavioural components that constrain swarm placement and spatial structure have yet to be defined. Whatever the mechanism involved, swarms and swarm clusters are weak points in the biology of the malaria mosquito that are ripe for exploitation.

#### Mechanisms of reproductive isolation

One of the crucial roles of swarms in the mating system of *An. gambiae* is to provide conspecific males and female with a mating arena in which they can select potential mates i.e. intra-specific sexual selection. However, swarms also play a key role in pre-mating reproductive isolation between the sibling species and forms. In the process of swarming, choosing potential mates, and leaving the swarm in copula, females and males are capable of accurately choosing conspecific mates. Understanding the processes involved in these steps is crucial because it could open the door to novel ways of attracting or trapping and killing males or females. Crucially, it can also bring about a much needed breakthrough in our understanding of the male mating phenotype, which could be the stepping stone for improved mating competitiveness and mating specificity of mass-produced males for release programmes.

##### Spatial swarm segregation

One of the better-characterized mechanisms of pre-mating reproductive isolation in complex populations of *An. gambiae* is the spatial segregation of swarms. Since the original description of partial swarm segregation between *An. coluzzii* and *An. gambiae* in villages in Burkina Faso [15], swarm segregation has been reported in Mali [6, 8] and amongst the sibling species, *An. melas* and *An. gambiae* s.s. in coastal Benin [10]. Evidence from these studies suggests that swarm segregation can serve as a first barrier to hybridisation amongst cryptic taxa but it is clearly not the sole determinant as evidenced by the occurrence of mixed swarms at various frequencies [10, 15]. Significant differences in the average height of the swarms have also been reported between sub-taxa and between populations of *An. gambiae* s.s. [10]. The onset and duration of swarming have been shown to be constrained by the timing of sunset and they largely overlap across the forms and sibling species of the *An. gambiae* complex [8, 16]. As a consequence they cannot constitute a major mechanism of reproductive isolation. The overall emerging picture is that spatial segregation of swarms plays a major role in the reproductive isolation of *An. gambiae* in inland West Africa. Whether or not the same picture holds in those western coastal regions of Africa where significant hybridization between *An. gambiae* and *An. coluzzii* have been reported remains to be seen [17]. Indeed, the ecological and genetic factors that promote the breakdown of reproductive isolation barriers between the two species in these environments remain largely unknown. Understanding those factors could inform mosquito release programmes seeking to use interspecific mating permissiveness in order to target several cryptic taxa simultaneously (see below).

##### Within-swarm conspecific recognition

Although spatial segregation is a major mechanism of reproductive isolation between the sub-taxa of *An. gambiae* it cannot solely explain it [8, 18]. The possibility that males produce aggregation pheromones that serve to attract females to swarms has repeatedly been explored without success [19]. In any case, the volatile nature of aggregation pheromones would make them unlikely mediators of conspecific recognition in the dynamic system that are swarms and mixed swarms in particular. Contact pheromones, on the other hand, could provide short-range cues necessary for conspecific recognition [19, 20]. Evidence from two mosquito species suggests that contact pheromones could play a role in the mating process. In the winter mosquito *Culiseta inornata,* males are thought to recognize conspecific emerging females through a pheromone present on the females’ legs thanks to receptors located on their own tarsi [21, 22]. In the tiger mosquito *Aedes albopictus,* males seemed to recognize conspecific females thanks again to receptors located on their tarsi [23]. Unfortunately, the lack of further publications on this topic speaks for itself. Attempts to identify consistent differences in cuticular hydrocarbon profiles between *An. coluzii* and *An. gambiae* have so far been inconclusive [24].

In the absence of any newer evidence for pheromone-based recognition in mosquitoes, research efforts have largely focused on flight tones as putative cues used in conspecific recognition within swarms. Whilst early reports suggested that significant differences in the frequency of wing-beat alone could account for conspecific recognition [25, 26], the largely overlapping wing-beat distributions of cryptic taxa of *An. gambiae* made for an unlikely isolation mechanism [27]. However, the hypothesis that flight tones produced in the process of courtship and mate choice may be key to conspecific recognition proved correct [27]. Studies conducted with *Toxorhynchites brevipalpis* [28, 29], *Aedes aegypti* [30], *Culex quinquefasciatus* [31] and *An. gambiae* [29] all showed that tethered female and male mosquitoes respond to one another’s flight tones and can adjust their wing-beat frequencies in such a way that it results in convergence of harmonic frequencies between the sexes (reviewed in [32]). Harmonic convergence does not occur between same-sexed individuals and is more likely to occur when a male and female are compatible, either because they belong to the same cryptic taxon [29] or because they are in the presence of a preferred mate. For females, preferred mates can be larger than average [33] or generally more attractive to them [34]. At present it is still unclear whether harmonic convergence occurs simply because a male and female that are initially attracted to one another attempt to meet in flight and copulate. If this is the case, the signal(s) responsible for the initial attraction remain unknown and could be qualitative flight tone differences indicative of size, vigour and cryptic taxa, or possibly other cues. Alternatively, harmonic convergence could play an integral part in close-range courtship flight and the resulting dynamics would decide if a potential mate is suitable or not. One study showed that free-flying *Ae. aegypti* males that induced harmonic convergence with tethered females also promoted higher female fecundity when mated to them [34]. In addition, their male progeny were more likely to induce harmonic convergence, suggesting a heritable component in the signal responsible for male attractiveness that is used in female choice [34]. It should be noted that the technical prowess required for recording flight tones has made recording harmonic convergence in the field very difficult [33]. However, it is hoped that recent progress in 3D video recording of swarming individuals, perhaps combined with flight tone recordings, may soon reveal the exact processes involved in mate choice.

### Targeting swarms for malaria control

One can distinguish two major types of interventions that directly depend on male mosquito mating behaviour and therefore rely heavily on our limited understanding of its processes. The first potential application is related to the development of sound or chemical traps that make use of putative sensory cues used by *An. gambiae* in swarm formation. Whilst several studies have highlighted the role of wing-beat frequency in mosquito mating, the role of chemical cues (contact or release pheromones) is less clear and deserves further investigation. The second potential intervention is the recently proposed lure-and-kill strategy exploiting visual cues involved in swarming.

#### Sound trap

That mosquitoes are attracted to the wing-beat sound produced by conspecifics has been reported since 1878 [35]. Ever since, entomologists have considered the possibility of controlling mosquitoes by means of sound traps. This idea became even more attractive with the realisation that, even though wing-beat frequencies were variable, they often overlapped among species [36]. It was thought that the overlapping in wing-beat sound frequency would be advantageous for attracting multiple species and both sexes of mosquitoes in the field. The first attempt to attract and control mosquito populations by a sound trap took place in Cuba in 1949 [35] when a large number of *An. albimanus* males were collected using a sound-baited trap. Further attempts at removing males succeeded in depressing female insemination rate in *C. tarsalis* [37] as well as significantly reducing the rate of parous females in *C. tritaeniorhynchus* [38]. From a practical point of view, two major issues need to be addressed to enable the potential use of this approach to tackle malaria vectors. First, there is a need to design a trap that can effectively attract and capture large numbers of male mosquitoes from a distance. Secondly, the traps would need to be placed strategically, which requires devising novel means of rapidly identifying clusters of swarms within intervention zones.

As far as sound trap design and specificity are concerned, the conclusion from mosquito wing-beat studies is that the fundamental flight-tone harmonic differs between females and males but often overlaps between closely-related species, thus complicating the design of species-specific traps [26]. Importantly, within swarms, mosquitoes are thought to communicate by means of much more complex flight-tone characteristics that occur at short range [28–30]. Therefore, one of the key priorities for trap designs would be to better describe key acoustic signals used in mate-choice within swarms and attempt to amplify them over greater distances. The exact effects of increased sound intensity on the performance of such flight-tone traps are currently hard to predict. However, in *Gryllus integer* [39] and *Scapteriscus acletus*, increasing sound intensity above natural levels generally increased their attractiveness [40, 41].

In previous studies mosquito sound traps were usually placed in the vicinity of larval breeding sites. This may not be optimal because male mosquitoes are not sexually mature when they emerge from breeding sites and may not be attracted to traps at all. In African malaria vectors, mating typically occurs away from breeding sites if these are located outside of the villages, and the position of swarms within villages often matches that of distinct swarm markers [6]. Therefore, a particularly interesting prospect would be to revisit the use sound traps in combination with that of visual cues such as swarm markers to target swarming individuals.

#### The Lure-and-kill strategy

**Fig. 3:**
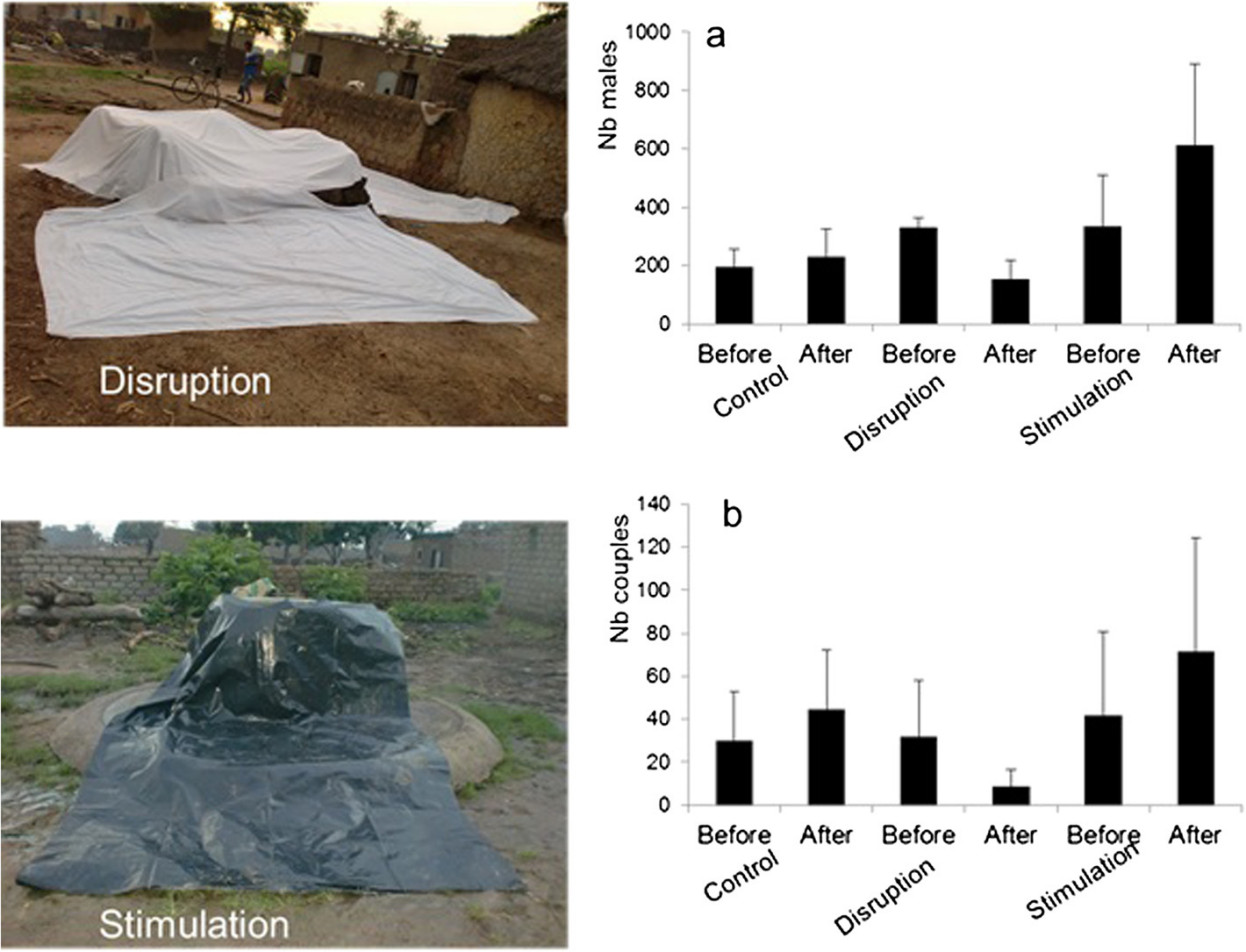
Manipulation of swarms of *An. gambiae* using white sheet (disruption) or black plastic sheet (stimulation) overlaid on swarm natural markers: (**a**) refers to the mean number of males per swarm while (**b**) refers to the number of mating events per swarm. Bars are standard errors of the mean

The second potential intervention tool targeting male mating behaviour is the lure-and-kill strategy that exploits swarm visual cues. Among all possible stimuli that draw male and female mosquitoes together over a swarming arena, visual cues seem to be the most important. Swarms that constantly occurred at the same sites were seen to disappear when the swarm markers were accidentally removed [6, 12]. Furthermore, some observations suggest that swarming can be artificially disrupted or enhanced through manipulating artificial markers. In a preliminary experiment conducted in the Kou Valley in 2010, black and white cloths were spread over the ground to attract or repel swarms (Fig. 3) (Toe, Dabire, Tripet F and Diabate unpublished). The number of males attending swarms and the mating events in each swarm were significantly reduced with the white cloth, while these numbers significantly increased with the dark cloth (Fig. 3). These observations suggest that swarms can be manipulated by means of visual cues and hence become easier targets. The strategy of lure-and-kill would therefore consist of creating a so-called kill zone within villages where males are attracted *en-masse* and killed. Around this zone, swarms would be disrupted to push them into the kill zone. In a preliminary experiment designed to assess the potential impact of swarm killing on *An. gambiae* populations, Diabate and colleagues were able to decrease mosquito densities by ~80 % in houses by targeting and mass killing swarms in the Valley de Kou (Sawadogo, Bilgo, Niang, Maiga, Dabire, Tripet and Diabate unpublished).

Successful deployment of the lure-and-kill strategy requires that swarms are easily identifiable, that they swarm constantly at the same place, that they are easily accessible and that most males in the populations are attracted to the kill zone. Over several years of investigations in Burkina Faso, Mali and Benin, swarms were recorded forming over the same markers year after year [6, 8]. Importantly local villagers were recruited on-site and successfully trained to identify and collect swarms, suggesting that a community-based approach could be used for swarm killing programmes [6, 8]. Such intervention, despite its apparent difficulties, has the advantage of targeting malaria vector species that are not affected by IRS and ITNs as well as outdoor-biting mosquito populations that currently escape conventional control tools.

### Use of swarms as mosquito population sampling tool

In the face of insecticide resistance, there is growing concern that malaria eradication will not be achieved without the introduction of novel tools. Improved SIT and genetically-modified (GM) male mosquito releases may play an important role in future integrated vector control or eradication programmes. Because they rely on the release of mass-reared males, the full potential of given SIT and GM programmes can only be assessed through measurements of critical male parameters, such as longevity, dispersal and mating competiveness. At present, we know very little about the resting sites of males and the classical entomological methods used to sample females in the field are not relevant to males. Currently, there are no standard and effective methods of sampling males to measure these parameters. In many regions, males are mostly found outdoors and can be sampled using Muirhead-Thomson pit shelters and other outdoor resting sites, but yields are often negligible. Estimation of such parameters would therefore require mark-release-recapture experiments but the rate of recapture is so low that some wonder if it is worth devoting such effort. The principal difficulty is that male mosquitoes are mostly found outdoors and during the rainy season typified by high mosquito densities, so although many outdoor resting sites are available, efficient sampling of males becomes difficult. Because male mosquitoes must gather in swarms to ensure their reproductive success, swarm sampling resolves the problem of having to hunt males in scattered resting sites and can therefore facilitate the evaluation of interventions relying on male mosquito releases.

It is noteworthy that the entomological tools currently used to evaluate the efficacy of interventions are highly ineffective when vector densities drop below a certain threshold. With the prospect of malaria elimination/eradication becoming a reality in some African countries comes the need for better tools to effectively monitor key parameters involved in malaria transmission, including effective methods of sampling of sparse endo and exophagic vector populations. The problem is similar to that of sampling dry season mosquito populations in seasonal habitats. Residual populations are reduced to such a point that catching females requires enormous sampling efforts. In contrast, surveying swarms at the same time period will generally still provide males in larger numbers. Assuming a 1:1 male to female ratio, it becomes evident that females must be present as well but cannot be reached with current collecting methods. A few novel female sampling devices have been developed over the last few years [42], including some that promise to perform as well or even better than traditional human landing catches [43, 44]. However, when these sampling tools reach their limit, swarm sampling could be used as a proxy measure of residual female population density. Repeated longitudinal sampling of a number of known swarm sites might thus be a very effective strategy for generating quantitative estimates of changes in vector population size.

### Mating behaviour and mosquito release programmes

Mosquito release programmes for vector population suppression or replacement constitute another category of interventions whose success directly depends on male mosquito mating behaviour and are therefore currently hindered by our limited understanding of its processes. These interventions include ‘classic’ releases of chemo or radio-sterilized males and modern approaches such as genetically-modified sterility-inducing males, *Wolbachia*-carrying males designed to suppress or replace populations through cytoplasmic incompatibility, and males with genetic drive mechanisms spreading effector genes into wild populations (reviewed in [45–47]).

The most obvious determinant of male mating competitiveness is their capacity to mate with wild individuals from the target population. Mating competitiveness can be tracked by examining the progeny of wild females captured after the releases in order to check if released and wild individuals mated randomly [48–50]. In the past, and despite the best efforts to maintain adequate phenotypic quality, non-random mating patterns have often been detected and held responsible for low effective mating ratios and the poor results of several mosquito release projects [50–53]. Given how little was and is currently known of the complex mosquito mating phenotype it is not surprising that hit or miss results are the best that can be hoped for [54]. Rearing techniques aiming at protecting the mosquito mating phenotype in the laboratory do not go further than the occasional genetic strain refreshing scheme and crude measurements of fitness. This is simply because we currently do not know precisely what phenotypic characteristics make for a sexually more attractive mosquito under natural swarm-like conditions. Nor do we know what makes a mosquito capable of differentiating conspecifics from other individuals in a mixed swarm. Given the complexity and size of anopheline malaria vector populations in Africa, these knowledge gaps may prove crucial for the cost effectiveness and success of release programmes.

#### The problem of assortative mating

##### Assortative mating amongst cryptic taxa

*An. gambiae*, with its vast geographical range and complex population structure, epitomizes the challenges associated with non-random mating for mosquito release programmes. The presence of sympatric cryptic taxa in many regions, combined with a limited knowledge of the processes leading to strict assortative mating amongst them [55] (see section 2), currently casts doubt on the feasibility of implementing successful release programmes [56]. Whilst the last decade has seen substantial progress in our knowledge of male mating biology [57], it has now become even more imperative to further our understanding of the environmental and genetic determinants of mating competitiveness and mating choosiness in wild populations.

**Fig. 4:**
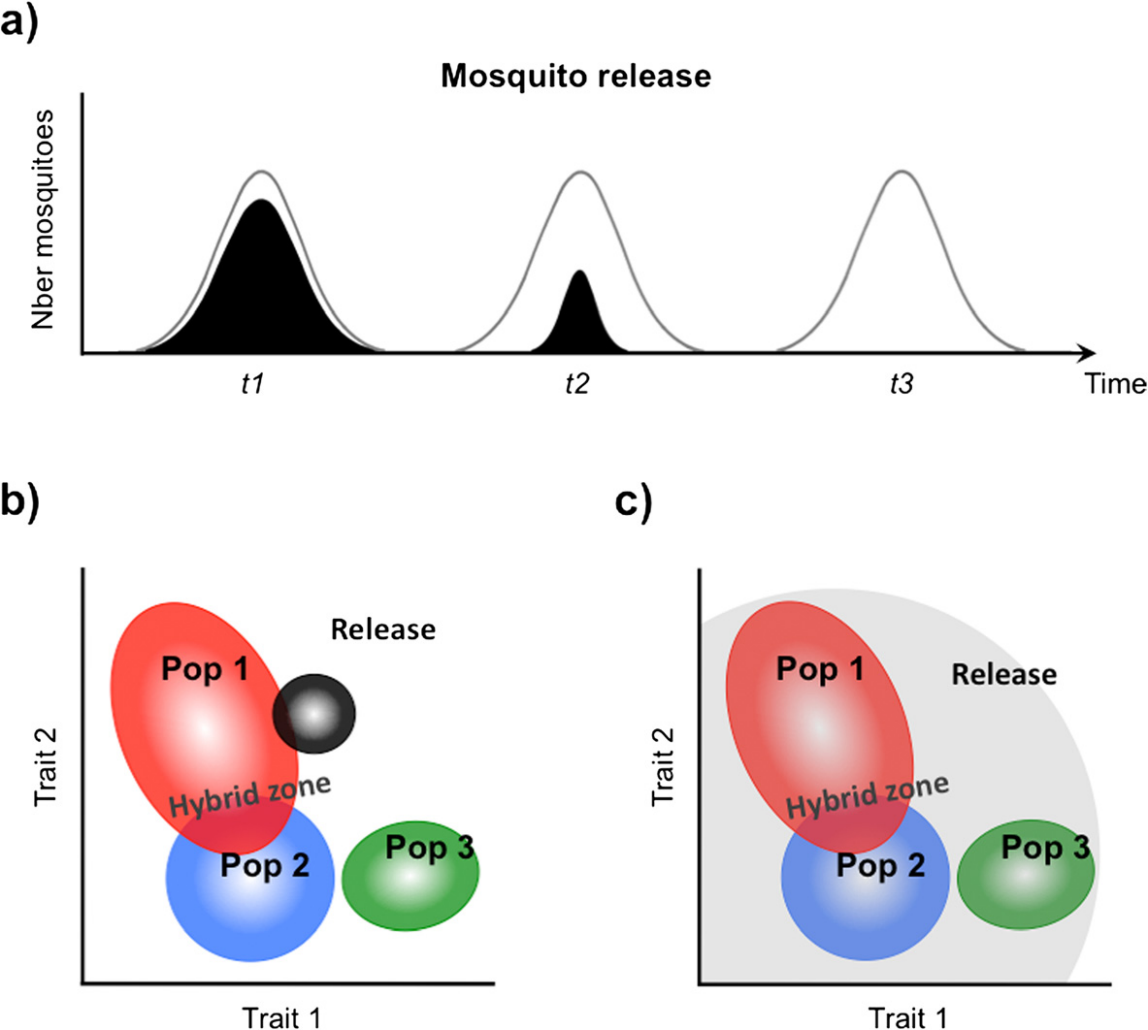
The success of mosquito releases depends heavily on the mating performance of released individuals: (**a**) ideally, the distribution of the mating phenotype of release individuals (white curve) exactly matches that of the target population (black curve) and repeated releases (time *t1*, *t2*, *t3*…) achieve population suppression. In (**b**) and (**c**) three sympatric sub-taxa of *An. gambiae* (red, blue and green) are characterized by distinct distributions (seen from above) in a hypothetical two-dimensional mating phenotype space (traits 1 & 2). In (**b**) the phenotypic distribution of the release strain (black) is narrower than that of population 1 it originates from, effectively decreasing the effective ratio of mating with wild individuals from population 1, there is no mating with other populations. In (**c**) environmental effects on the development of adult mating behaviour prevent mate discrimination in released individuals (grey shade) and mating occurs at low rates with any of the cryptic taxa present in the area

One of the consequences of strong assortative mating amongst cryptic taxa for mosquito release programmes conducted on a broad geographical scale, is that locally-derived strains will need to be developed for each sub-taxa to ensure effective mating (Fig. 4a,b). This may be relatively easy for release programmes relying on radio or chemo-sterilized males since the genetic background of the strains they are produced from can be maintained by re-colonization and/or regular outcrossing with field-caught individuals. By comparison, improving the mating phenotype of genetically-modified strains (whether sterile or carrying effector genes) is crucial yet much more complicated. This is first and foremost because transgenic lines are often produced from inbred laboratory-adapted lines whose mating phenotype differs considerably from that of recently colonized or field-caught individuals [54, 58]. Secondly, and this may vary according to the gene drive and genetic construct(s) considered [46, 59], strain refreshment will often require elaborate crossing and selection steps to achieve transgene homozygosity. Independently of the fitness costs they might impose on laboratory lines [60, 61], transgenic constructs may also directly affect mating performance in the field by interacting with one or several genetic pathways critical for mate choice. It follows that strain refreshment schemes would greatly benefit from a better understanding of the genetic and environmental determinants of assortative mating in order to produce males that effectively mate with their intended target populations [62]. The same knowledge could potentially lead to the production of males, generally competitive and attractive to females and thus capable of mating indiscriminately. This approach may be advantageous in targeting multiple populations co-occurring in a single ecological zone [62] and particularly effective in areas of natural hybridizations between populations (Fig. 4a,b).

##### Size assortative mating

**Fig. 5:**
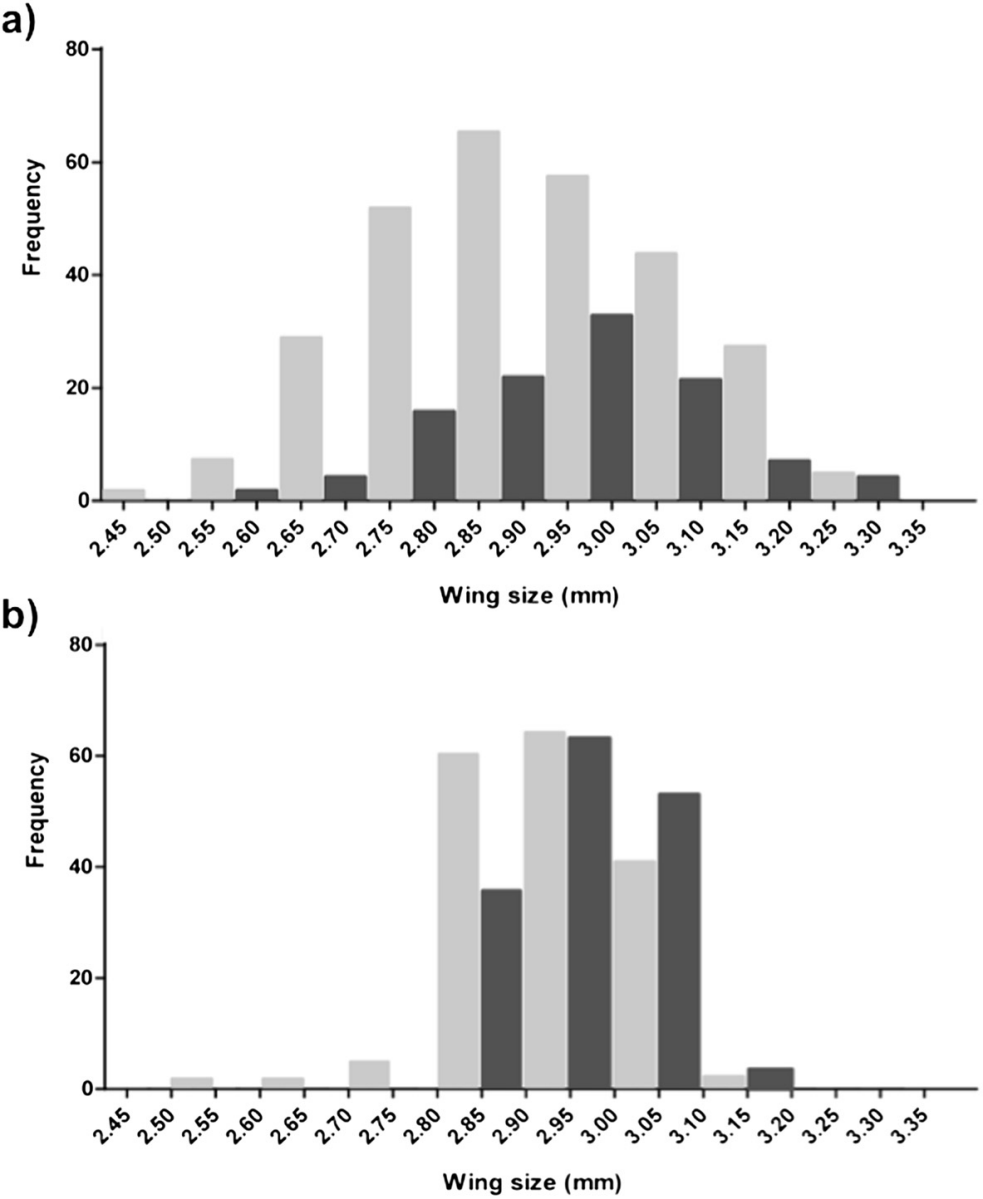
Evidence that body size correlates with mating attractiveness in *An. gambiae*: In (**a**) mated males captured in swarms are usually larger than unmated ones (modified from Maiga *et al.* [65]); in (**b**) larger females are favoured by individual males in a laboratory choice experiment (Ekechukwu and Tripet*,* unpublished data). Note the narrower phenotypic distribution of laboratory-reared individuals

Size-assortative mating is another potential hurdle for mass-production and releases of *An. gambiae*. In mosquitoes, a larger body size is often equated with higher phenotypic quality because of the known causal cascade between larval growth conditions, imaginal size, and subsequent survival and desiccation resistance [63, 64]. Regardless of the cryptic taxa they belong to, there is now strong evidence that females do not simply prefer to mate with larger males. In field studies of natural swarms conducted over several years and different locations in Burkina Faso, males caught in copula were repeatedly found to have significantly larger mean body size than that of other swarming males [16, 65] but with a size distribution centred around an intermediate-to-large body size [16, 65] (Fig. 5a). Laboratory experiments that experimentally manipulated male size have reported similar results, with mated males typically being larger than unmated ones and intermediate size males securing the majority of copula [63]. These results suggest that intermediate-sized males mate more successfully either because they are more agile in flight or because they can make contact with females faster or maintain contact with females for longer amounts of time within swarms [63, 66]. There is currently no evidence that male body size affects the overall time that males spend in natural swarms [16]. Neither does it seem that larger males could secure more mating because of sheer body strength. Optimal male body size is thought to be under stabilizing selection because of the trade-off between selection for smaller and more agile body sizes associated with mate choice in swarms and the need for survival and desiccation resistance [16, 63]. In addition, females may actively be selecting males of an intermediate size over others, in which case female preference would be another force contributing to stabilizing selection on male size. It is noteworthy that, under warmer and wetter climates, selection for desiccation resistance and hence larger body size may be weaker, leading to a lower optimal and preferred male mating size. This could explain the smaller body size and lack of difference in body size of resting, swarming and copulating males observed in a study conducted on São Tomé Island, Equatorial Guinea [12]. The development of 3D video recording technology allowing for detailed video tracking of swarms promises to shed light on the processes of sexual selection. Early results suggest that females approach several males before a successful pair is formed thereby suggesting elaborate mate selection processes [13].

The possibility that males exert mate choosiness and sexual selection on females has been largely unexplored. Swarms create conditions in which male competition for females is high and reproductive success could be largely driven by female choice. Such lek-like conditions typically lead to very skewed distributions of male reproductive success, with males of higher phenotypic quality securing most of the copulations [6, 8]. Under laboratory conditions and with ample supplies of sugar and water, anopheline males are thought capable of inseminating up to five females per night [67]. Therefore, the best males may have the opportunity to inseminate several females over a few days, display some mate choosiness, and impose sexual selection on females [68]. In *An. gambiae*, as in most insects, above a threshold, female fecundity rapidly increases proportionally with body size [69, 70] and this could explain why males were found to preferentially mate with females from a larger-sized strain over smaller ones in group mating experiments [70]. Individual males also choose larger than average females when given a choice of mating partners (Ekechukwu and Tripet, unpublished) (Fig. 5b). Taken together, these data suggest that males may actively choose larger females in order to maximize their reproductive success.

**Fig. 6:**
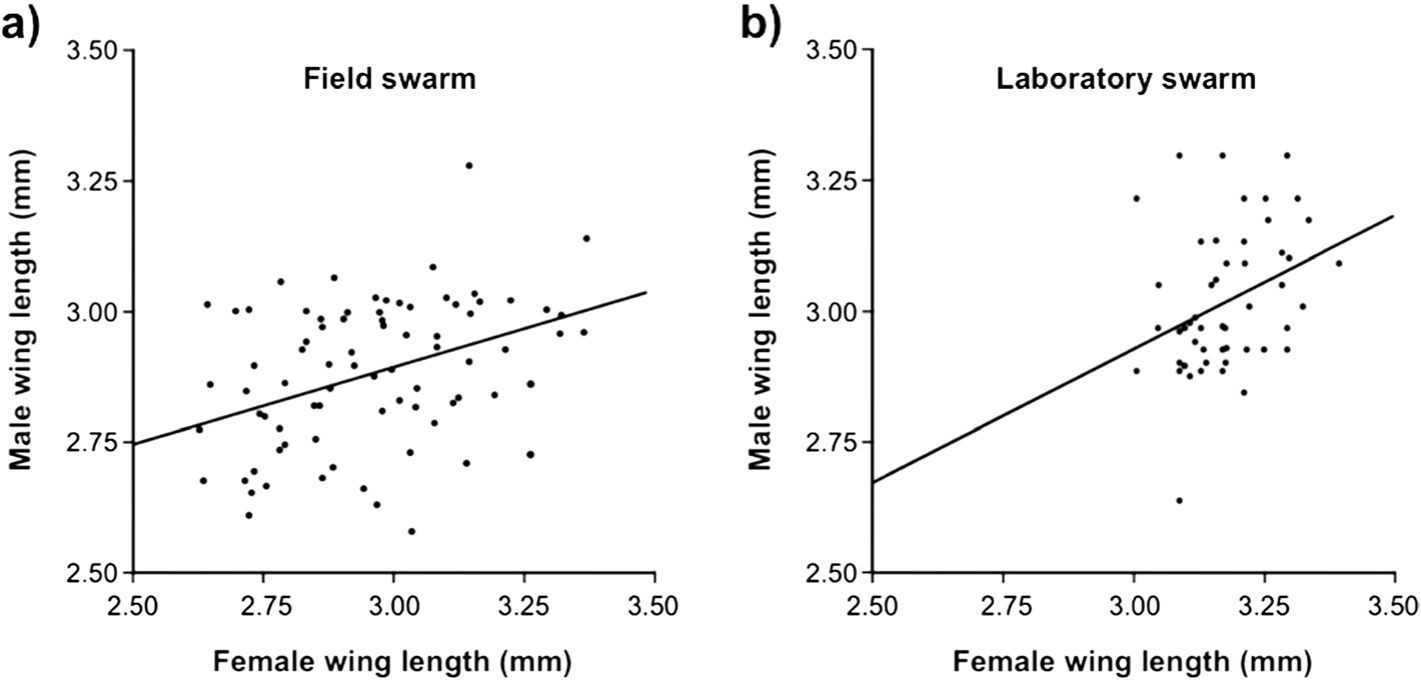
Size assortative mating in swarms implies that, in terms of mating, larger is not simply better and that releases should aim to produce a broad distribution of mosquito phenotypes. In (**a**) size assortative mating in wild swarms of *An. gambiae* (modified from [65]); and (**b**) in laboratory produced swarms of an old laboratory strain (Ekechukwu and Tripet, unpublished data)

Importantly, field studies have found that the body sizes of male and female *An. gambiae* captured *in copula* are often correlated [65]. The correlation is weak to modest and has also been observed under laboratory conditions (Ekechukwu and Tripet, unpublished) (Fig. 6a,b). The occurrence of size-assortative mating could explain the low kurtosis of the distribution of mated males in swarms compared to unmated ones. The possibility that too large a size difference might negatively affect mating success by impacting the flight of pairs *in copula*, successful sperm and mating plug transfer, or simply the time spent in tandem hence predation risks, has not yet been explored. Any of these reasons, could result in only a limited number of potential mates being considered in the swarm whose size range would not fully reflect the size distribution of the whole swarm.

Since vector control programmes based on the release of sterile or genetically-modified mosquitoes rely so crucially on release ratios and effective mating ratios, understanding what makes a mosquito sexually attractive is paramount [4, 58]. In a recent paper Maiga *et al.* [65] were the first attempting to predict male attractiveness using a measure of their fluctuating asymmetry in wing length rather than simply body size. The level of symmetry in the length of the left and right wing, which is thought to be a possible cue used by females to assess male quality, was quantified. However, no significant difference in this parameter was found between mated and unmated males [65].

##### Environmental determinants of assortative mating

Non-random mating between insectary-reared and wild mosquitoes may also arise because of known or unknown differences in environmental conditions between rearing facilities and natural breeding sites. In what has become a classic example, a difference in the photoperiod used for mass-production of sterile male *An. culicifacies* carrying a complex chromosomal aberration prevented males from finding and inseminating wild females [71, 72]. Paton *et al.* [61] recently evaluated the carry-over effects of larval rearing environment on adult mating competitiveness and mating choosiness in *An. coluzzii* using field-cages in Mali*.* The progeny of field-caught females and females from a strain colonized from the same location were reared in a field insectary exposed to outdoor conditions or in an indoor insectary with otherwise comparable density, feeding regimes, rearing trays and access to natural photoperiod *via* a glass wall. The results of this study showed that laboratory-strain progeny reared in the indoor insectary mated competitively but were unable to choose mates of their own cryptic taxa. Interestingly, the same laboratory-strain progeny reared in the field insectary mated assortatively [62].

It is clear that most problems linked to the mating performance of mass-reared strains [58, 72] could potentially be avoided if enclosures reproducing natural-like environmental selection pressures on mating behaviour were designed [73]. Although mosquito mass-rearing has been rendered more practical by the use of artificial membrane blood-feeders and other technical improvements [74–77], most of those improvements have resulted in higher numbers of mosquitoes being produced but were traded against the behavioural requirements of the strains [73, 78]. Space is commonly not a limiting factor in malaria-endemic countries and, provided that adequate funding is available, new techniques will need to be designed that combine efficient mosquito rearing with the maintenance of normal mating behaviour.

#### Evolution of behavioural resistance to mosquito release

**Fig. 7:**
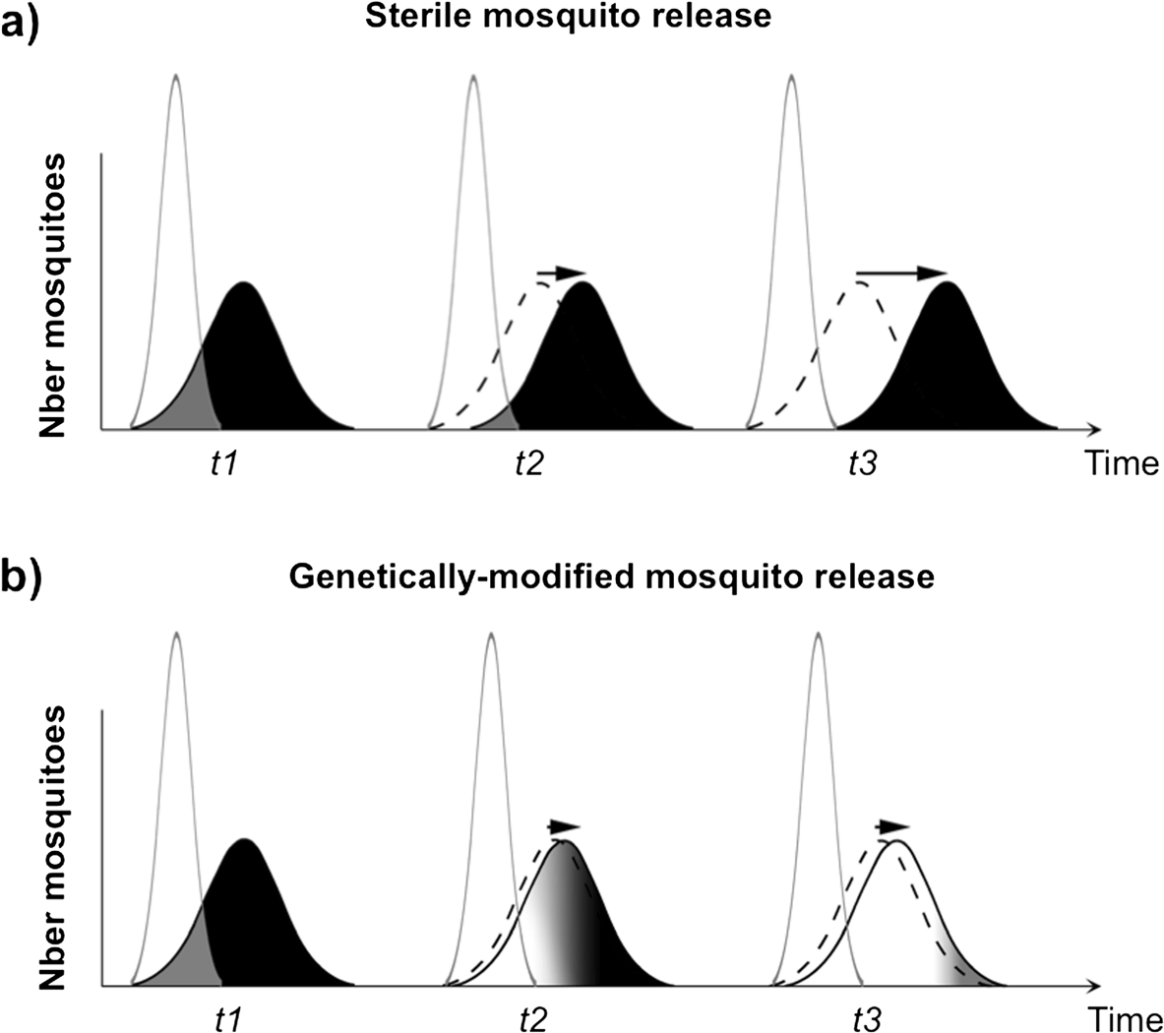
The repeated release of mass-reared individuals with a narrower distribution of mating phenotype due to decreased genetic and/or phenotypic variance can drive the evolution of mating avoidance in target populations. In (**a**) the repeated mass releases (time *t1*, *t2*, *t3*) of sterile individuals whose narrower mating phenotype distribution only partially matches that of wild individuals imposes hard selection on the target population and drives the evolution of pre-mating isolation barriers. In (**b**) the gene drive mechanism implicit to most genetically-modified mosquito releases limits the opportunity for the evolution of mating avoidance in target populations resulting in successful target population transformation (change from black to white)

Another potential hurdle inherent to repeated releases of sterile individuals is that, in spite of preventive measures taken to maintain their genetic and phenotypic qualities, the target population may evolve mechanisms to avoid mating with the release strains. This problem was clearly stated by Huettel [73] who considered that: ‘In essence, the male sterile technique is an exercise in post-mating reproductive isolation’. In effect, any long-term release of unfit mosquitoes could result in speciation-like processes that foster pre-mating reproductive barriers between released and wild individuals. In theory, the same phenomenon could potentially occur in genetically-modified mosquito releases, provided that the genetic construct bears a fitness cost to its carriers and simultaneously modifies their mating phenotype such that they can potentially be discriminated against by non-modified individuals. The use of an efficient gene drive mechanism and strains specific to each target sub-population may help pre-empt such pitfalls (Fig. 7a,b).

Divergence of mating behaviour between target and release strains may have been partly responsible for the decrease in male competitiveness seen in many sterile male releases. Previously, these have been attributed to inadequate rearing conditions [72]. Evolution of mating avoidance was also postulated by some as the reason for the progressive failure of the early screw-worm eradication programme in Northern United States [79]. Divergence of mating behaviour was also revealed in studies following failures of the melon fly eradication programme in Japan [80] and from a pilot eradication programme targeting Mediterranean fruit flies in Hawaii [81]. Such divergence of mating behaviour has, however, never been adequately quantified in mosquito release programmes. Aside from these studies, little data is available to understand what factors facilitate the evolution of mating avoidance. Initial differences in the mean and variance of key traits determining mating success would certainly increase its likelihood. Mating avoidance is also more likely in long-term release programmes with inadequate strain refreshing, as well as those programmes targeting poorly described wild populations with unknown genetic and ecological heterogeneities.

## Conclusions

By all accounts, research on malaria vectors has reached a golden age over the last two decades. New molecular tools have been made available to investigate the whole breadth of insecticide resistance mechanisms that currently hamper vector control in the field. Against the backdrop of failing pesticide efficacy, the astounding progress of molecular biology and genomics have fuelled hopes that new vector control approaches based on the release of better SIT or GM males to control malaria will be available soon. Thanks to an abundance of new genetic markers, we also have a much better understanding of the complex structure of vector populations in Africa and of patterns of gene flow amongst them.

Whilst most established areas of research have directly benefitted from post-genomic progress, comparatively little progress has been made in identifying and studying new mosquito phenotypes that can be exploited for vector control. One such phenotype is male mating biology. As we have shown in this paper, a better knowledge on male biology can be instrumental to a range of potential interventions. These include crucial improvements of approaches based on male releases for the control of field populations. A better knowledge of male swarming and mating biology can also open new territories for vector control, such as those focusing on swarm-generated sounds for use in traps or those focusing on the development of swarm manipulations and killing approaches. Finally, swarms can be used as a proxy to measure female densities in areas and periods where conventional sampling tools fail thereby facilitating the monitoring of future interventions.

Ecological studies are a first fundamental step towards translational studies that can exploit these novel possibilities. They should also pave the way for integrative behavioural and molecular ecological studies thereby effectively bridging the gap between ecology and genomics as has been the case in the past with other phenotypes crucial to malaria transmission.
